# European Core Health Indicators - status and perspectives

**DOI:** 10.1186/s13690-018-0298-9

**Published:** 2018-08-03

**Authors:** Angela Fehr, Mariken J. Tijhuis, Sabrina Hense, Dominika Urbanski, Peter Achterberg, Thomas Ziese

**Affiliations:** 1 Department of Epidemiology and Health Monitoring (Dept. 2) Unit 24 – Health Reporting, Robert Koch-Institute (RKI); (SH until June 2017), P.O. Box 650261, D-13302 Berlin, Germany; 2 Centre for Health Knowledge Integration, National Institute for Public Health and the Environment (RIVM), PO Box 1, 3720 BA Bilthoven, The Netherlands

**Keywords:** (3–10): ECHI, Policy relevance, Data availability, Comparability, Health information

## Abstract

**Background:**

The European Core Health Indicators (ECHI) are a key source of comparable health information for the European Union (EU) and its Member States (MS). The ECHI shortlist contains 88 indicators which were developed by experts from MS and international organisations. Most indicators are derived from data sources at the EU’s statistical office (Eurostat), the World Health Organisation (WHO) and the Organisation for Economic Co-operation and Development (OECD) and are available for most MS. The remaining indicators on the shortlist are at different stages of conceptual and/or methodological development. The indicators have been reviewed in the past against scientific developments, changes in data collections and emerging policy needs, yet not as part of a systematic and sustainable procedure. There is also no regular inventory of problems met by the MS in collecting the necessary data. Work package 4 of the BRIDGE Health project aimed at updating and improving the existing ECHI-indicator knowledge and expertise and at strengthening the scientific base that supports the effective development and use of health indicators for health policy evaluation and prioritization by the EU and its MS. The aim of this paper is to present a first overview of its outcomes and to explore issues concerning the ECHI data availability, content and policy relevance, update process and accessibility to stakeholders, in light of working towards a sustainable future.

**Methods:**

Two surveys were conducted within the framework of the BRIDGE Health project to reassess the status of the ECHI shortlist. The first survey focused on data availability in EU MS, candidate countries and European Free Trade Association (EFTA) countries. The second survey evaluated current needs and criteria with respect to content and policy relevance of the ECHI shortlist. Exploring potential new indicator topics was part of both surveys. All evaluations were supported by an advisory network of national and international experts.

**Results:**

Of the 36 countries (EU MS, candidate and EFTA countries) contacted for the data availability mapping, 23 countries (63%) participated in the survey. Data availability from preferred data sources varied between chapters. Availability was highest for the chapter on demography and socio-economic situation, followed by the chapter on health status, where data were available for most indicators from more than 90% of the participating countries. Problems experienced by MS relating to the incorporation of ECHI into their health systems were also identified through the survey. Findings from the survey on policy relevance point at the need for strengthening the links with policy (priorities) and for exploring a possible format change of the list to accommodate actionability. It also showed support for embedding ECHI in a sustainable health information structure; this may practically be aided by a web-based single point of access to an information repository.

**Conclusion:**

Policy relevance is an essential but not systematically developed criterion for the inclusion of indicators into the ECHI shortlist. Data availability is crucial for the actual implementation of indicators and has considerably increased for ECHI in the last decade. The data availability mapping provides a structured overview of the current status of data availability for implemented indicators. The ECHI shortlist can contribute to the collection of comparable policy-relevant health data in Europe, foster evidence-based public health and contribute to Member States learning from each other. Flexible and systematic incorporation of policy relevance in the ECHI shortlist review and revision process may substantiate ECHI as a core component of a future sustainable European health information infrastructure.

**Electronic supplementary material:**

The online version of this article (10.1186/s13690-018-0298-9) contains supplementary material, which is available to authorized users.

## Background

**Fig. 1:**
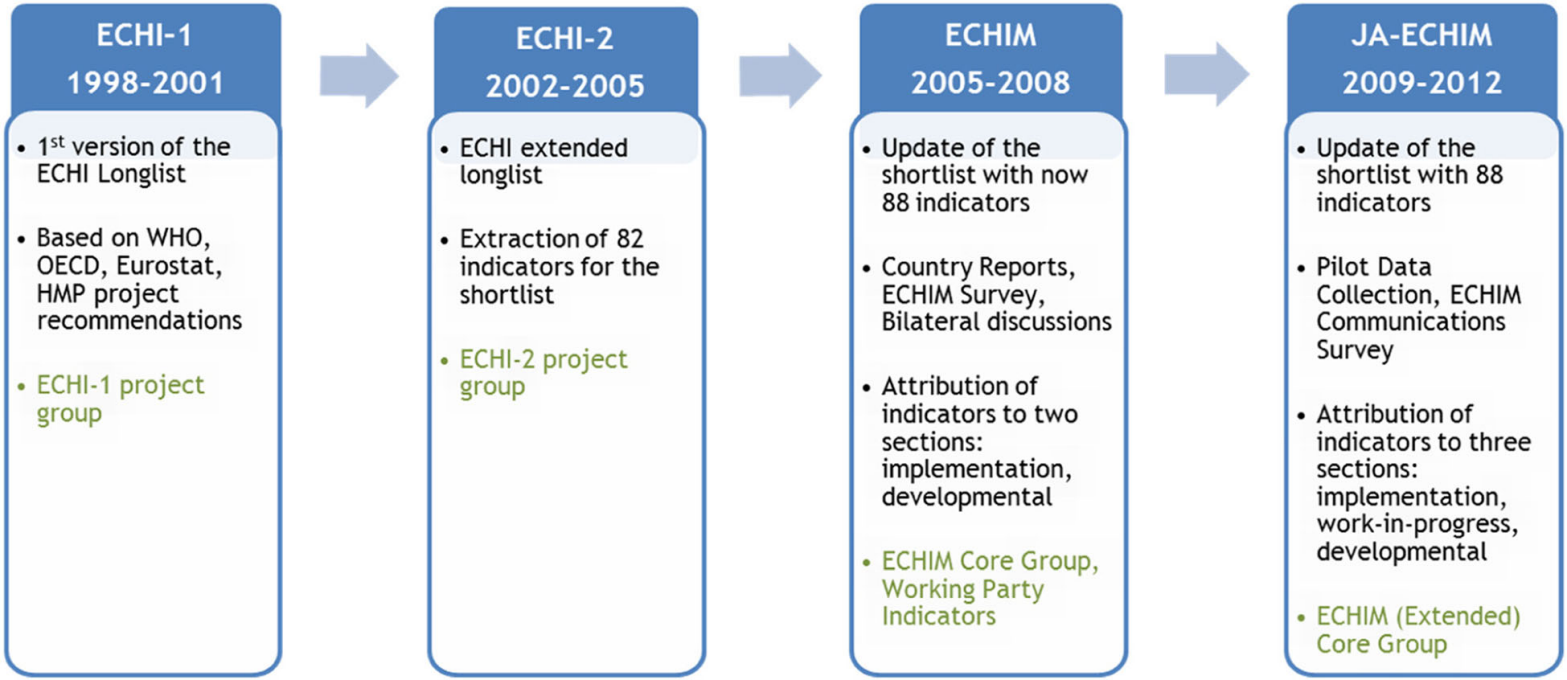
History of the ECHI process. In 4 consecutive projects, covering the years 1998–2012, the ECHI Shortlist was developed and its implementation was initiated. At the end of this period, the list contained 88 indicators, 67 of which were ready for implementation, 14 were close to ready and 13 were not (at all) ready. ECHI: European Core Health Indicators, known as European Community Indicators before 2013; ECHIM: European Community Indicators Monitoring; JA-ECHIM: Joint Action for ECHIM

Comprehensive, targeted and valid health information is essential to monitor population health and guide policies aimed at protecting it [1]. In 1997, the EU established a Community action programme on health monitoring, one key objective being the development of comparable Community health indicators [2]. In four consecutive EU-funded projects (ECHI-1, ECHI-2, ECHIM, Joint Action (JA)-ECHIM; see Fig. 1), involving MS and international organisations and covering the years 1998–2012, a core set of 88 public health indicators (“ECHI shortlist”) was developed and its implementation in the health monitoring systems of MS was initiated [1, 3–5].

The initial indicators’ longlist was developed in close collaboration with projects run under the EU Health Monitoring Programme [6]; the shortlist was then selected and prioritized by a group of public health experts [7]. The shortlist was designed to monitor trends in population health and its determinants but not to be static per se. It was agreed that in order to maintain the relevance and stability of a comprehensive health information system over the years to come, new indicators would need to be added and older ones deleted. In this process, public health relevance and practical data availability were both considered relevant, but basically different dimensions. It was therefore recommended not to mix these in the same selection procedure [7], but to select on policy-relevance criteria first, and establish the data availability and the precise indicator definition after that. This was expected to avoid the trap of data being driven by availability, but nonetheless result in generally good data availability. At the same time it would expose for which (relevant) indicators a need for data development may still appear.

ECHIM suggested a selection procedure in which policy relevance as well as practical feasibility of a predefined list of indicators was rated by the Working Party Indicators [8]. The JA-ECHIM then developed a new procedure and formulated clear criteria for selecting indicators (Table 1) as well as for possible necessary adaptions (Tables 2 and 4) [1].

**Table 1:** Criteria for the selection of ECHI shortlist indicators [20]

i. The list should cover the entire public health field, following the commonly applied structure of the well-known Lalonde model: health status, determinants of health, health interventions/ health services, and socio-economic and demographic factors.	
ii. The indicators should serve the user’s needs, meaning that they should support potential policy action, both at the EU and MS level.	
iii. Existing indicator systems, such as the indicators used in the WHO Health for All database and OECD Health Data, should be used as much as possible, but there is room for innovation.	
iv. Use the viewpoint of the general public health official (‘cockpit’) as frame of reference.	
v. Focus on the large public health problems, including health inequalities.	
vi. Focus on the best possibilities for effective policy action.

**Table 2:** Eligibility criteria for the three sections of the ECHI shortlist, also used for section transfers [20]

Eligibility criteria for the implementation section (currently 67 indicators)	
There is consensus on the indicator definition and calculation, and data are adequately available in international databases -- > the indicator can be used to support policy making, it is ready for implementation at (inter)national level	
Eligibility criteria for the work-in-progress section (currently 14 indicators)	
There is consensus on the indicator definition and calculation, or considerable developmental work has already been carried out (i.e. consensus can be reached within a limited amount of time), but the indicator is not yet incorporated in regular international data collections. There is an overview of national data availability and data are available in a reasonable number of countries -- > technically, the indicator is (nearly) ready for incorporation in regular international data collections, but there may not yet be concrete plans for this.	
Eligibility for the development section (currently 13 indicators)	
This section contains those indicator topics that are not ready yet for incorporation in international regular data collections (and thus for implementation) due to considerable methodological and/or data availability problems.	

The JA-ECHIM finalized for each indicator an overview of all the information needed for computing the indicator (the so-called documentation sheet), including the definition, the data source and type to be preferably used, a description of data availability and relevant policy area/s. The current shortlist indicators are grouped into five main chapters: Demographic and socio-economic situation (9 indicators); Health status (32 indicators); Health determinants (14 indicators); Health services (29 indicators); and Health promotion (4 indicators). Within the documentation sheets, the indicators are mapped to 17 (non-exclusive) policy areas. The European Commission (EC) has further condensed these to 12 minor and 5 major policy areas: Health services and health care; Ageing and population; Health determinants; Diseases and mental health; and Health in all policies [9, 10].

Indicators on the shortlist derive their data from a variety of sources, including Eurostat, the WHO’s European ‘Health for all’ database (HFA-DB) or the database of the OECD. Data types encompass official statistics, survey and administrative data. Important survey data come from the EU-Statistics on Income and Living Conditions (EU-SILC) and from the European Health Interview Survey (EHIS). Additionally, indicators such as on tobacco, alcohol and drug consumption, on accidents or on environmental monitoring, use international, topic-specific reporting systems [1, 11]. The indicators on the shortlist are allocated to three sections, reflecting the different degrees of implementation readiness, as defined by the JA-ECHIM (Table 2). One criterion for inclusion in the implementation section is adequate data availability. By the end of the JA-ECHIM in 2012, half of the member states had incorporated the ECHI into their national health information system, and several more stated that they were in the process of doing so [4].

Ongoing important challenges for ECHI are how to accommodate scientific developments, changes in data collections and emerging policy needs as part of a systematic and sustainable procedure that will serve health monitoring and policy making in EU and MS. The JA-ECHIM advised to review the ECHI shortlist on a regular basis, and issued additional recommendations for future indicator work (Table 3). Consequently, the BRIDGE Health project (2015–2017), aiming to develop a sustainable EU health information system for public health and research purposes, included the ECHI in its activities. Its work package (WP) 4 was tasked with evaluating, updating and improving the existing ECHI shortlist, taking into account previous evaluations and other BRIDGE Health work packages. WP4-activities were jointly carried out by the Robert Koch Institute (RKI) in Germany and the National Institute for Public Health and the Environment (RIVM) in the Netherlands.

**Table 3:** JA- ECHIM- Summary of recommendations for future indicator work [20]

i. Ensure sustainability, quality and efficiency of the ECHI indicator work	
ii. Keep the ECHI indicator documentation up to date and easily accessible	
iii. Work with supra/international organisations and Member States on further harmonization of existing data collections	
iv. Work on improving implementation-readiness of indicators in the work-in-progress and development section	
v. Update the ECHI shortlist on a regular basis.	

The aim of this paper is to present the first results of these activities, which focused on data availability, policy relevance (adequacy and flexibility) and on the needs for a transparent and sustainable ECHI process. The outcomes may serve as a starting point for further work towards a sustainable future for the ECHI.

## Methods

In order to map the data availability for the ECHI and evaluate the policy relevance of the shortlist, we developed two surveys and established two expert groups. The following section describes the composition of the two expert groups as well as the surveys’ background and design.

### Involvement of expert groups

To support its activities and to strengthen and maintain the network of national and international health information experts, WP4 established two experts groups:

- An Advisory Core Group (ACG), comprising representatives of international organisations (Eurostat, OECD, WHO) and/or of academia in the field of public health. This group was asked to provide strategic direction to the work of WP4, ensuring that its activities align well with developments at European and international levels.
- An Expert Group on National Health Indicator Implementation (EG-NHII) consisting of over 20 members of the EU Expert Group on Health Information (EGHI; https://ec.europa.eu/health/indicators_data/eghi_en). Its main task was to assist WP4 in identifying issues surrounding the national use and implementation of ECHI-indicators.

### Data availability survey: development and implementation

Several data mapping exercises with different foci have been carried out in the framework of ECHI(M) projects [4, 12, 13]. Between 2005 and 2008, the ECHIM project explored data availability in international and project databases as well as in national sources [8]. The JA-ECHIM (2009–2012) performed a Pilot Data Collection to “obtain comparable data for 20 ECHI shortlist indicators that were unavailable or incomparable in these international databases” [1]. The latter exercise resulted in a more refined shortlist and in comprehensive documentation sheets for each indicator, covering a broad spectrum of relevant metadata (e.g. definition, policy area and relevance, preferred data sources and data types, calculation). Also, the shortlist sections were expanded from previously two to three sections (implementation, work in progress, developmental). Building on these previous exercises, the objective of the WP4 availability survey was to explore data availability for the ECHI in the relevant preferred data sources and data types. Furthermore, the survey aimed to identify potential adaptation needs for the ECHI Shortlist that may have arisen since its last update in 2012. Progress or a decrease in data availability was to be explored, as well as the availability in countries that recently joined and/ or had not been part of an earlier exercise. To collect the information, excel sheets were developed, pre-filled, where possible, with country-specific information on data availability in preferred data sources and types, and sent to national contacts with the request to confirm or amend the information. An attempt was also made to gather information on possible new indicator topics by asking the survey participants to name topics for which, to their knowledge, national data needs existed. The participants were encouraged to provide full-text comments and, concluding the survey, to fill in a section on their background, affiliation and ECHI-related experience. A draft of the survey and of the accompanying guidelines for participants was pre-tested in a group of experts on international indicators. In April 2016, the survey was sent out to experts from 36 EU MS, candidate and EFTA countries; participants were asked to respond by June 2016. Within this timeline, two reminders were sent to non-responders.

### Content evaluation survey: development and implementation

The ECHI content survey was developed taking account of previous evaluations and with the aim to serve future demands and development of the shortlist. We specifically mention here the 2013 external evaluation of the use and impact of ECHI, commissioned by the European Commission, which concluded that increasing the usefulness for policy planners should become a priority (see Table 5). If the list develops towards being more of a policy instrument, addressing evolving information needs of policy makers and steering the strategic policy planning and monitoring process across Europe, this would have implications for the ECHI shortlist size, flexibility and balance. Hence, these aspects were included in the survey.

The central question of the survey was: how can we improve the current policy focus, balance and appropriateness of the ECHI indicator approach to better serve stakeholders?

It consisted of 3 parts:

- Respondent background and affiliation
- Shortlist criteria, flexibility, size, balance, policy relevance and utility
- Support in identifying literature in which ECHI are used or evaluated

The survey was created in an online form management system (https://en.formdesk.com/) and accessible via a link sent by email. Pausing and resuming without loss of data was made possible.

Questions were formulated variably in open and closed (checkbox and radio) format.

The survey was first piloted with the ACG (see section on involvement of expert groups) in February 2017 and adapted according to feedback. It was then launched with the Members of EGHI (*n* = 50), with an option to forward to others, in March 2017. Completion was requested in April; reminders to non-responders were sent twice. Final results were received in May 2017.

The survey’s main findings were presented and discussed in a face-to-face expert meeting in May 2017 with members of EG-NHII and ACG, and interested BRIDGE Health leaders/representatives.

## Results

Below, we present findings from the surveys on data availability and on policy relevance.

### Data availability survey

#### Survey participation

Of the 36 countries contacted, 23 (63%) responded to the survey, 21 being EU MS.

The majority of participating experts (9) were affiliated with a national public health institute, followed by employees of health ministries (7), of other employers (4; e.g. National Health Information Center, Center for Disease Prevention and Control, Diabetes Register, National Board of Health and Welfare) and statistical offices (2). Sixteen respondents were members of the EGHI group and 20 were in addition/instead involved in other international activities regarding health indicators. These included activities organised by Eurostat, WHO, OECD and the European Monitoring Centre for Drugs and Drug Addiction (EMCDDA), as well as the Joint Assessment Framework on Health (JAF Health)/Indicators Subgroup of the Social Protection Committee, the Technical Assistance and Information Exchange Instrument of the European Commission (TAIEX), EURO-PERISTAT and the Study on Health Behaviour in School Aged Children (HBSC). Some surveys were jointly answered by several experts; therefore, the number of affiliations exceeded the number of participating countries.

#### Summary of outcomes

**Fig. 2:**
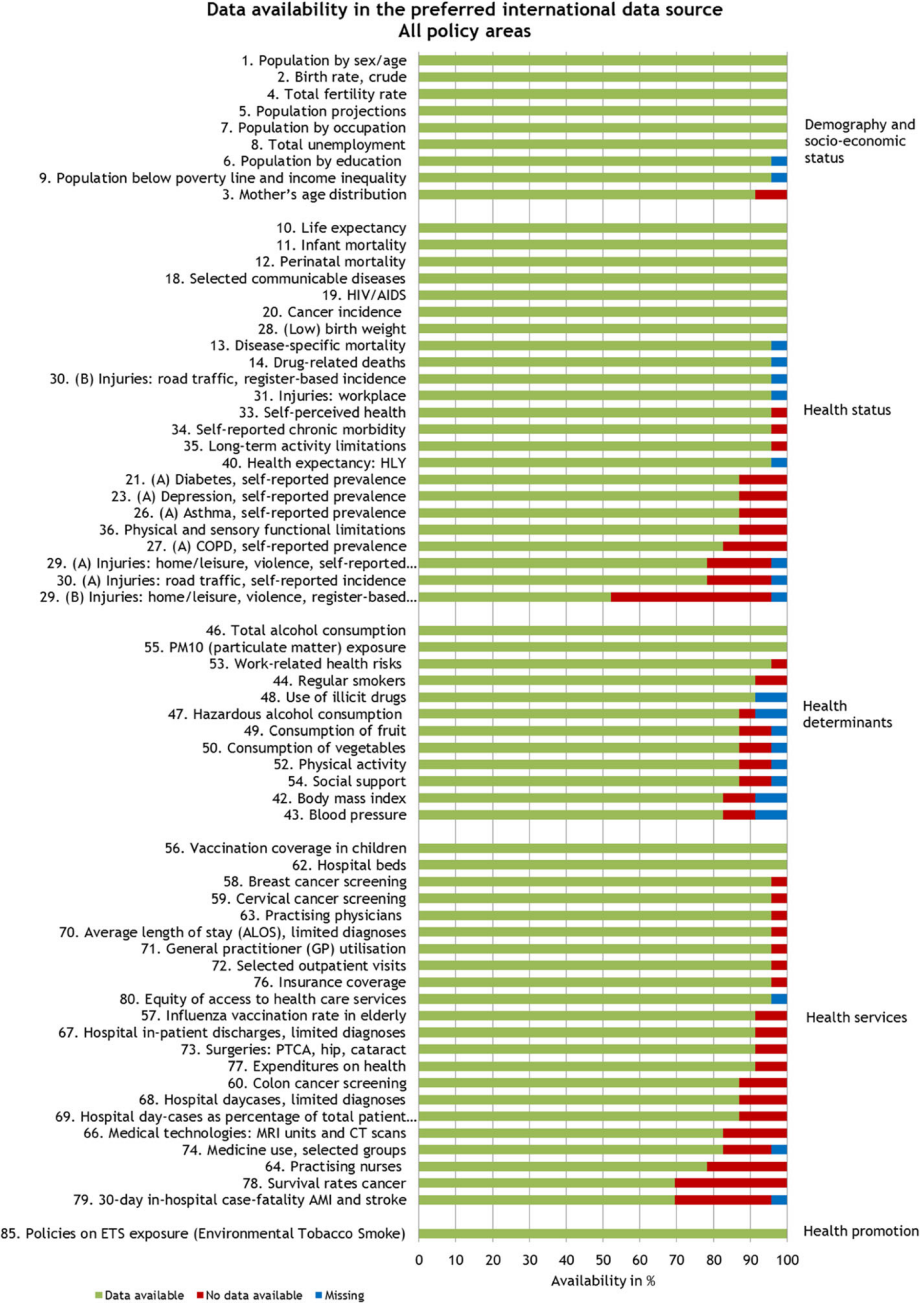
Proportion of countries with available data in the preferred international data source (implementation section/*n* = 67). Of the 88 indicators on the ECHI shortlist, 67 are in the implementation section. For most of these indicators, at the time of collection in April–June 2016, data was available from the preferred international data source for at least 75% of the countries participating in the survey

**Fig. 3:**
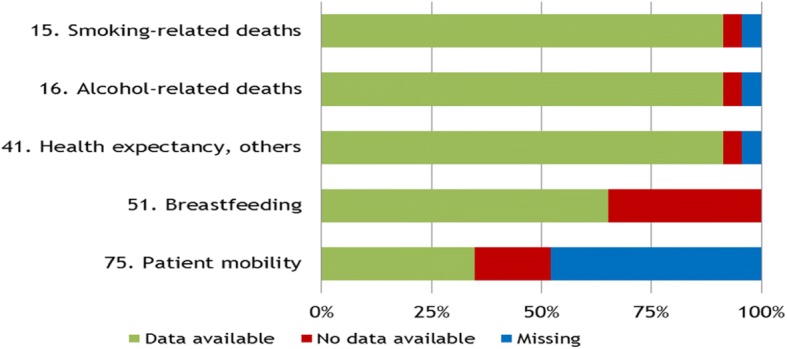
Data availability for work-in-progress indicators with defined preferred international data sources. In the work-in-progress section (*n* = 14), only 5 indicators had a defined preferred international data source, of which only the indicators 15 (Smoking-related deaths), 16 (Alcohol-related deaths) and 41 (Health expectancy, others) had data available from the preferred international data source in more than 75% of the participating countries

The analysis of the survey responses was performed using different perspectives: By section (implementation, work-in-progress); by preferred data source / preferred data type, and by main chapters of the ECHI shortlist (demography and socioeconomic situation / health status/ determinants of health / health services / health promotion). Figures 2, 3 below show the data availability in the preferred international data sources for indicators in the implementation and the work-in-progress section. Percentages for both figures can be found in Additional file 1: Tables S1 and S2.

In the implementation section, for the chapter “demography and socio-economic situation”, data is available for all participating countries for most of the indicators; exceptions were mother’s age distribution (not available in one EU member state and in the candidate country) and population by education as well as population below poverty line and income inequality (due to missing replies for the candidate country). Indicators on “health status” were assessed for prevalence as well as for incidence/mortality, where applicable. For most indicators in the area on “health status”, data is available from more than 90% of the participating countries. Exceptions were some indicators for which the EHIS is the preferred international data source; however, except for those indicators where derogations were granted to MS with respect to the transmission of data [14], EHIS indicators will shortly be available from all EU MS. The lowest availability (52%) was reported for injuries: home/leisure, violence, register-based incidence. Full availability of data in the policy area “determinants of health” is given only for total alcohol consumption and PM10 [particulate matter] exposure. Some indicators are based on the EHIS and thus should by now have become available. The availability of indicators on “health services” is close to or over 90% for about half of the indicators in this policy area and below 80% for practicing nurses, survival rates cancer and 30-day in-hospital case-fatality Acute Myocardial Infarction (AMI) and stroke. In the “health promotion” area, availability was assessed for policies on environmental tobacco smoke; this indicator is available for all participating countries. The remaining indicators in this area are developmental indicators with no preferred data source yet.

For five of the 14 indicators in the work-in-progress section, a preferred international data source has already been defined. For three of them (smoking-related deaths, alcohol-related deaths and health expectancy, others), the availability is above 75%. (Fig. 3) Four of the remaining nine indicators in this section have no defined international data sources yet (24. AMI; 25. Stroke; 45. Pregnant women smoking; 61; Timing of first antenatal visits among pregnant women). For five indicators (21. (B) Diabetes, register-based prevalence; 23. (B) Depression, register-based prevalence; 26. (B), Asthma, register- based prevalence; 27 (B) COPD, register-based prevalence), Eurostat’s diagnosis-specific morbidity data shall be the preferred international data source.

In addition to mapping data availability for implemented and work-in-progress indicators, the survey explored data needs for indicators in the development section and for possible new indicator topics, including topics suggested by survey participants. The survey results show that data on several topics of the development section is judged important by more than 50% of the responding MS. This refers to data on psychological distress, on psychological well-being, on suicide attempt, on waiting times for elective surgeries and on surgical wound infections. Responses to this section of the survey were limited, however, with missing rates between 22 and 43%. Possible new topics outside of the development section that seemed to generate an interest in data are healthy ageing, disability and current depressiveness. Only 3 out of 23 participating countries suggested new topics; ‘caesarean section’ was proposed by two countries, with EURO-PERISTAT / national data as the preferred data source.

In a concluding section of the survey, the participants were asked to indicate whether regular meetings on the implementation of ECHI indicators (e.g. in national implementation teams) take place in their countries and whether general data problems regarding health information exist at the national level in their countries. Five respondents reported that regular meetings on the implementation of ECHI indicators take place in their country. General data problems with regard to health information were reported by seven countries; they comprise aspects such as: the ICD-10 being in place for mortality, but not yet for morbidity, the linkage between different databases being limited, responsibilities for health information being fragmented throughout the country, the sample sizes of international surveys being too high for small countries, and the decrease in government funding for (public health) statistics as a risk for deteriorating quality and quantity of health statistics and health monitoring.

### Content evaluation survey

#### Survey participation

Twenty experts contributed to the survey, representing a total of *n* = 18 countries, with broad geographical coverage. Combined, they were knowledgeable of all public health areas, some being generalists and some with expertise in one or more specific areas, most often morbidity/disability and mortality. About half of the respondents were affiliated with a government structure and about half with a (science-based governmental) public health institute. About half characterized their work as bridging between science and policy, about a quarter as relating most to policy and a quarter as relating most to science. As far as tasks within the policy cycle, *n* = 15 were involved in monitoring and forecasting, and *n* = 12 in benchmarking, and *n* = 5 were involved in health system performance assessment, target-setting and policy evaluation each.

#### Summary of outcomes

The results below provide a summary of the views of the survey respondents unless otherwise specified (e.g., some of the experts present during the final face-to-face meeting had not filled out the survey but did contribute to the discussion).

##### Criteria for selection, addition and deletion of indicators

The selection criteria used to compose the current shortlist (Table 1) were considered relevant up to this date. However, there were some suggestions for different wording, e.g. to include health system performance under the scope of public health (criterion i).

The criteria for additions (Table 4) were generally considered relevant (the criteria each being agreed on by 90–100% of the respondents), but some suggestions for rewording were put forward. For example, the importance of the issue (criterion i, on policy relevance) *should* not *(*but *may* be) reflected by its appearance in leading policy documents; indicators should also be able to serve an agenda*-setting *function by promoting the uptake of an issue into leading policy documents. In addition, in the definition of policy relevance, next to possibilities for *prevention* also possibilities for *intervention* could be taken up.

**Table 4:** Criteria for additions and deletions [20]

Criteria for additions	
i. “The indicator should have clear policy relevance. This implies that it should be related to one of the major public health issues in Europe, and the importance of the issue should be reflected by its appearance in leading policy documents. A public health issue is a policy relevant issue when it is linked to a high burden of disease, clear possibilities for prevention, and/or clear possibilities for reducing health inequalities”.	
ii. “The indicator should not disturb the balance of the ECHI shortlist, i.e. there should not be too many (overlapping) indicators for similar topics, and not too many indicators for ‘minor’ or contextual topics in the shortlist”.	
iii. “In line with the general goals and concepts underlying the ECHI shortlist, the shortlist should provide a ‘snapshot’ of public health from the point of view of the public health generalist”.	
iv. “In line with the general goals and concepts underlying the ECHI shortlist, the indicators in the shortlist should be suitable for providing a benchmark for reflecting time trends”.	
v. “In line with the general goals and concepts underlying the ECHI shortlist, the indicators in the shortlist should be suitable for providing a benchmark for international (EU) comparisons”.	
Criterion for deletions	
i. “The indicator is related to a topic that is no longer policy relevant”.	

The criterion for deletions (Table 4) was considered relevant, but considered to require further specification; also, other criteria may be added, e.g., ‘a new and better indicator has been identified for the same concept’, or ‘there is lack of between-country differences’.

##### Balance, redundancies and new topics

The criteria for additions state that the indicator should not disturb the balance of the shortlist by including too many indicators for similar topics or for ‘minor’ or contextual topics. This may seem self-evident, but it does not mean balance is a major goal in itself. Especially if policy relevance is considered a driver of the ECHI list, then this may justify taking up more indicators under the same priority theme as well as omitting some topics that are not considered relevant.

Several indicators and operationalisations were considered redundant, but only by a few experts each. They may serve as a signal, but are not further elaborated upon here.

The experts were also asked if indicators or themes were missing or *under*represented, both in open format and additionally by presenting them with a checkbox list of topics that had been collected in the availability survey. The options from the pre-defined list that were most frequently checked were ‘health inequalities’ (*n* = 9), ‘healthy ageing’ (*n* = 8) and ‘food and nutrition’ (*n* = 7); the open format yielded more diverse results. In the end, ‘a structured procedure is needed to identify new areas of policy information needs in the central indicator set’; out of *n* = 20 experts, *n* = 11 agreed and *n* = 8 strongly agreed with this statement (n = 1 had no opinion).

In addition, the idea was expressed to use ECHI as a pointer to other sets/collections, to allow for a more complete picture of a topic and enable ECHI to be more integrated in a ‘system’ of indicator sets across the EU. Examples given were pointing to the System of Health Accounts for health expenditure and pointing to Eurostat instead of having 86 causes of mortality under ECHI.

##### Flexibility/actionability

For a wider use and usability of the ECHI in the EU MS, the ECHI shortlist needs to be a recognizable brand. This would suggest that some form of stability of the list is critical. At the same time, relevant new issues may emerge and the shortlist needs to be sufficiently flexible to address these.

A mixed picture emerged from statements addressing this seeming contradiction. Out of *n* = 20 experts, *n* = 13 agreed and *n* = 1 strongly agreed to the statement that ‘stability is more important than flexibility’ and *n* = 6 disagreed; in addition *n* = 9 agreed and *n* = 2 strongly agreed to the statement that ‘it is important that ECHI indicators can indicate changes over a relatively short period of time’, whereas *n* = 8 disagreed.

A change in format may remedy this and accommodate the dual usage. The experts agreed on the need to investigate the option of changing the ECHI format to capture emerging information needs, for example by distinguishing different sections. Out of *n* = 20 experts, 7 agreed and *n* = 10 strongly agreed that ‘the ECHI list would benefit from establishing a stable core section and a flexible additional section to capture emerging information needs’ (*n* = 2 disagreed and *n* = 1 had no opinion). Another option for a format change, discussed during the final expert meeting, would be to use a form of layering such as developed under the Sustainable Development Strategy (SDS) Indicator framework [15] and adapted under the BRIDGE Health WP on Evaluation of health care systems for their european Health System Indicator (euHS_I) survey. This framework distinguishes indicators on 4 levels: headline, operational, explanatory and contextual. A related idea, raised in the survey, was the use of a top list of indicators (action -oriented), providing access to more detailed layers of information when needed (more analytic).

##### Size

The current number of indicators for all sections together is *n* = 88 (or *n* = 94, when counting separately those indicators that are based on both survey - and register data). These are actually representing a total of > 1000 operationalisations. Almost all experts considered the current number of indicators satisfactory for the ECHI shortlist but about half thought the number of operationalisations could be reduced. Reason for this is not solely there being too many, but also the difficulty to obtain some of the required disaggregations. It has to be noted that operationalisations in themselves were also considered very useful.

**Fig. 4:**
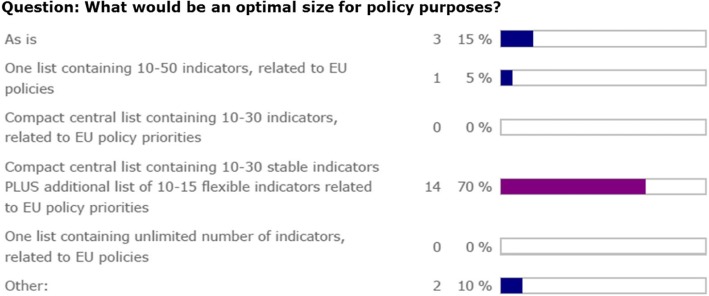
Expert opinion on pre-defined size options for an improvement of the ECHI list. A combination of a compact stable core and an additional flexible and policy relevant part was chosen as an optimal size by the majority of experts

However, for policy purposes, most agree that a different format, consisting of a compact stable core and an additional flexible part would be more optimal (see Fig. 4 below and related suggestions under ‘balance’ and ‘flexibility/actionability’).

One of the suggestions for the open format ‘other’ option was: a “Compact central list containing *30-50* stable indicators PLUS additional list of 10-15 flexible indicators related to EU policy priorities”.

##### Relevance and use

In the survey, the ECHI indicators were generally seen as policy relevant. The experts were asked to indicate which indicators had particularly low and high relevance and expressed concrete ideas on individual indicator’s relevance. Reasons given for attributing ‘low’ policy relevance to an indicator were that

- a better indicator is available (e.g. update from PM10 to PM2.5 - which interestingly has already been processed in the ECHI tool),
- it is very unspecific (e.g. lifestyle policies and integrated programmes in settings),
- its interpretation is unclear (e.g., is it better to have more hospital beds?), or that
- it is too specific (e.g., excess mortality by extreme temperature).

Quite a few indicators were considered highly relevant by at least some experts. To name a few that were reported by at least 5 experts and also emerged as particularly relevant in a previous evaluation [16]: 10.Life expectancy; 13.Disease-specific mortality; 20.Cancer incidence; 42.Body mass index; 44.Regular smoking; 56.Vaccination coverage in children and 77.Expenditure on health. In addition, the current survey’s top 10 also included: 21B.Diabetes; 40.Healthy Life Years; 52.Physical activity and 80.Equity of access to health care services.

However, it seems necessary to ask policy makers’ opinions to elaborate on this further, as well as to create consensus on what defines policy relevance and what its role should be in the ECHI list.

In the survey, the experts were asked for examples of documents in which ECHI indicators are used, documents that have specifically evaluated ECHI use or documents that provide examples of national policy making by using ECHI. There were some, but not many, examples of ECHI policy relevance or use in policy. There were no suggestions for documents that specifically evaluate ECHI use. We will not elaborate on this here.

The experts were also asked how the utility of ECHI could be advanced. The following sums up the goals that were considered necessary:

- A clearer link to policies and policy options
- Better and more visible links to other indicator and data sets (ECHI as part of a broader system)
- Better visibility of ECHI
  - for health policy makers
  - for society
- More active and formal approach to national entities

Some of the instruments that were suggested towards these goals were, among others:

- The use of policy targets and policy evaluation
- Regular ECHI-based reports (for different audiences, e.g. policy maker, researcher, society), involving also Parliament and the Directorate General for Health and Food Safety (DG SANTE), for Employment, Social Affairs & Inclusion (DG EMPL) and for Research and Innovation (DG RTD)
- Active recommendations to use ECHI and how to use them (a “for dummies” meta-dataset).

##### Development and implementation of an information repository

The experts provided many recommendations concerning the presentation and explanation of the ECHI indicators, which relates to aspects of accessibility and dissemination. During the final meeting, the experts discussed the concept of an ECHI information repository, which was presented to them as a single point of access aimed at a sustainable future, creating ECHI memory and possibly expanding towards including interactive facilities to exchange expertise and build capacity. The experts welcomed the concept of a web space where everything comes together; this web space could also include the idea of a pointer function towards other international organisations and projects, to avoid the time consuming task of collecting their meta-data or data (as has been part of previous projects). Technical aspects still need to be thought through, for example, the use of open source software and web publication principles.

## Discussion

The ECHI shortlist is the EU core set of public health indicators. It has been in use since 2005 and is the result of joint EU broad efforts in various projects since 1998, involving MS and international organisations.

In this paper, we explored the current status and future prospects for ECHI data availability and policy relevance using two surveys as well as additional expert sessions. Below, we discuss their outcomes in light of recent and historical developments and expectations related to the ECHI shortlist. The main issues concerned are the changing and improving data availability, the further development of the ECHI shortlist and its meta-data, its policy relevance and the need for an ECHI-update procedure.

### ECHI-data availability

**Fig. 5:**
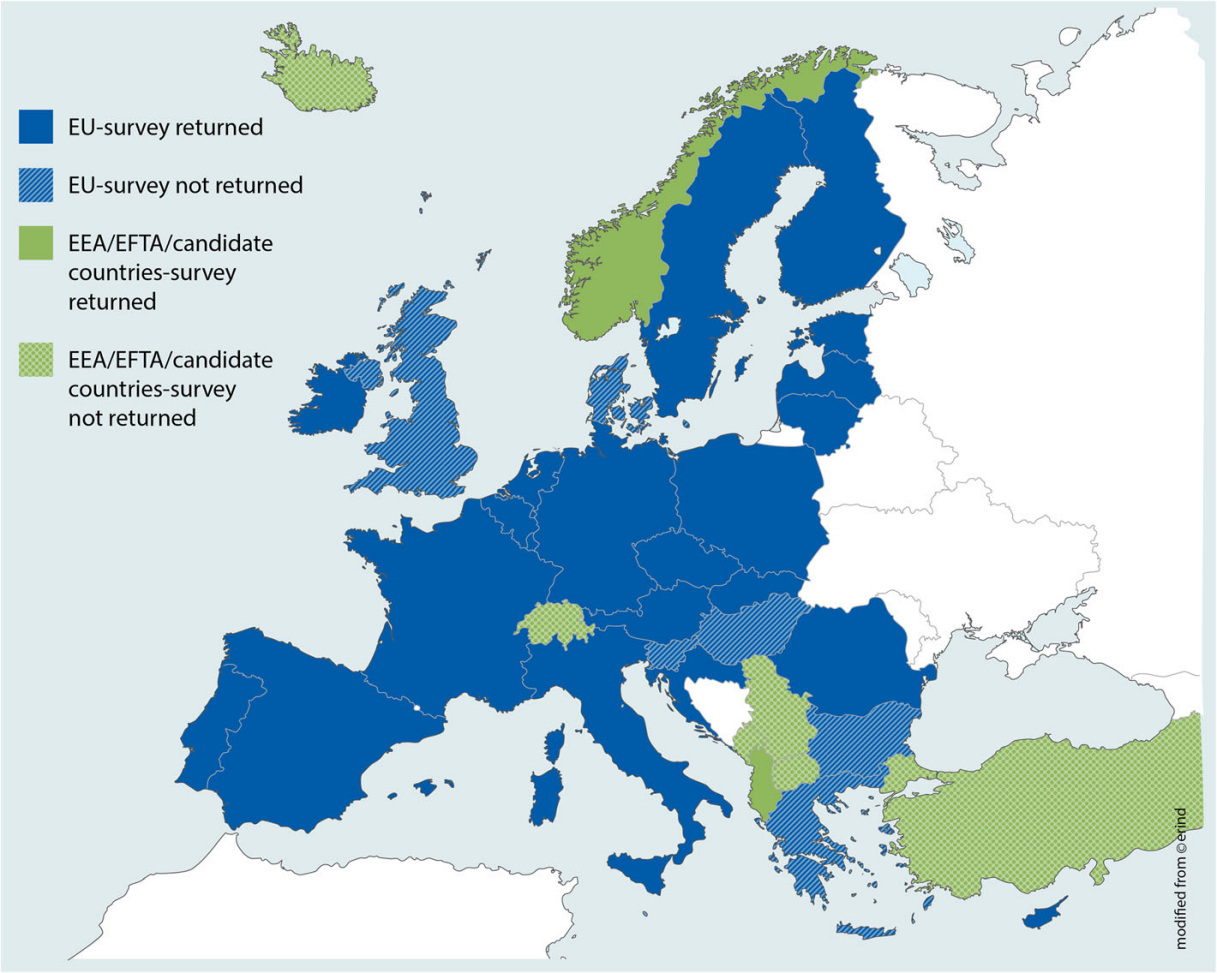
Survey participation “Data availability mapping”. National contacts of 36 countries (28 EU member states, 5 candidate and 3 EFTA countries) were invited to participate in the ECHI-indicator availability survey. The survey was returned by 21/28 EU member states, by one EFTA and one candidate country. In total, participating countries were: AL, AT, BE, CY, CZ, DE, EE, ES, FI, FR, HR, IE IT, LT, LU, LV, NL, NO, PL, PT, RO, SK, SE

The data availability survey provides us with a structured overview of the status of data availability and development needs for the ECHI shortlist in 21 EU MS, one EFTA and one candidate country. Of the 36 countries contacted, two countries declined their participation for lack of resources or lack of data. The remaining 11 countries did not respond to the initial invitation and to the reminders. It can be seen in Fig. 5 that the non-responding countries do not seem to cluster in one specific geographical area. We had hoped for a higher response rate but may have to conclude that responses and analyses could perhaps have benefitted from the use of an online survey tool. To ensure adequate selection of contacts for the availability survey, the most current membership list of the EGHI was used to approach potential survey participants. Also, EGHI members were encouraged to share the survey with national experts so that the relevant broad expertise could be used in filling it in.

Data availability varies between the main chapters of the ECHI shortlist with the chapter on demography and socio-economic situation being the one with the highest proportion of available data, followed by the section on health status.

The project presented here aimed to exclusively assess data availability for preferred data sources and types. Participants could, however, give additional information about other data sources and types available for ECHI indicators. This would inform about possible alternative data sources, should a country indicate that data were not available in the preferred source. Earlier ECHI data availability surveys applied different methodologies and pursued different objectives. Therefore, valid full and detailed comparisons regarding data availability (trends) between our survey and these studies cannot be made. Still, there are some general developments that have increased data availability and that allow to draw conclusions. Significant developments have taken place especially in the last decade regarding data availability for the ECHI. The EHIS, whose first wave (2006–2009) was only conducted on the basis of a gentlemen’s agreement, became mandatory for all EU MS as of its second wave (2013–2015). This has clearly increased data availability for the currently over 20 indicators which derive their data from the EHIS. Developments such as the European morbidity statistics project at Eurostat [17] may further increase data availability in the coming years [18] whereby the current shortlist of morbidity indicators may not be the final one, and some of the ECHI indicators may not be included in the shortened morbidity indicators’ shortlist.

New data sources may have to be identified for the ECHI shortlist and assessed for their potential of becoming a preferred ECHI data source. Only few of the 14 indicators in the work-in-progress section have preferred data sources or data types yet; these have to be elaborated. The survey also found discrepancies between the here reported data availability and availability reported from other sources for the same indicators which needs to be further explored. In addition, no work has been done to expand the list of preferred data sources for the work-in-progress section of ECHI.

### ECHI and policy relevance

So far, much of the earlier focus on improving and updating the ECHI shortlist has been on ensuring data comparability and overtime stability for broad health descriptive purposes; evaluation of ECHI content and policy relevance in particular has received less attention. Hence, recommendations for maintenance or improvement of the list have largely been made in relation to technical (data) issues, such as implementation-readiness and quality of data (see Table 3). These aspects are extremely important, but if it is considered important that ECHI takes on more of a steering role in health policy planning and monitoring, then other aspects need to be addressed as well, most notably the indicators’ policy relevance (see Table 5 for recent recommendations for use of ECHI and indicators in general). Our content evaluation survey confirmed previous and identified new ideas and views, which can be used to build a future for ECHI. It has to be noted that our survey results are based on a sample of health information experts; it now needs to be enriched with the views of policy makers. In this, we advise to take a broad perspective on public health and include health system performance (cf. [19]) under the scope.

**Table 5:** Recommendations from other evaluations

Recommendations report PHEIAC on ECHI use and impact (2013)	
• Minor modifications of the ECHI shortlist are possible.	
• Simplification / streamlining of the shortlist may be considered.	
• ECHI legal status should be clarified.	
• There is a need for increasing ECHI awareness among certain categories of policymakers.	
• The work-in-progress section of ECHI should be finalised.	
• Cross-country benchmarking should be encouraged.	
• It should become a priority to increase the usefulness for policy planners.	
• Address financing issues.	
Recommendations report Economisti Associati on EU Health Information System (2017)	
• Enhance the consolidation and coordination trends (in the larger European Health Information landscape).	
• Enhance policy-related use of harmonised indicators.	
• Adopt incremental measures to mitigate the burden of indicators.	

Examples of concrete use of the ECHI shortlist (and resulting policy lessons) has not been overwhelming, as concluded by the Public Health Evaluation and Impact Assessment Consortium (PHEIAC) [16], and does not seem to have improved much over the past years [This project]. On the other hand, use of ECHI indicators may be concealed by not referring to them as ECHI and it may be worthwhile to further investigate both implicit and explicit use. Still, both the ECHI and the EHIS play a normative role by guiding the selection and development of indicators in national health information systems. Additional evaluations are needed to see whether ECHI visibility and recognition is still poor in the formal policymaking process (i.e., staff responsible for planning and monitoring of policies or for policy evaluation and the assessment of healthcare services), as opposed to health information services, as was concluded by PHEIAC [16]. This also applies to their finding that uptake in general strategies and planning documents was poor, as opposed to benchmarking reports. In addition, it will be valuable to take into account the use of and possible overlap with indicators in other international sets, e.g., the recent indicator list developed by the Indicator Sub Group of the Social Protection Committee (JAF Health indicators).

At the time of the writing this paper, an evaluation by Economisti Associati, by order of the EC, was published on the cost/benefit balance of a sustainable EU Health Information System [18]. It concluded that enhancing policy-related use is a key issue for improving the benefits of having EU harmonised health indicators in place. Enhancing policy-related use could be supported by e.g. more policy-oriented “knowledge-based” products complementing the provision of indicators with analysis and adequate visibility and communication actions. These recommendations are along the same lines as our findings, where terms as policy relevance, flexibility and visibility played key roles.

Another recommendation included more flexible and rapid processes to update the indicators collected in view of emerging policy-relevant challenges.

### ECHI update procedures

During the ECHIM project (2006–2008), a procedure had been put into place by which working parties suggested new or revisions for indicators; indicator projects proposed and tested indicators, disseminated information, and the overarching Working Party Indicators ensured that indicator development was in line with needs of European health information and knowledge (voting procedure to select indicators). The JA-ECHIM (2009–2012) formulated clear criteria for additions and removals of indicators and for section eligibility, allowing the ECHIM secretariat to prepare substantial proposals and to compare countries’ suggestions against criteria. Expert involvement in the JA ECHIM differed from previous projects insofar as the focus was shifted from health information experts to MS representatives, taking account of the fact that the ECHI work moved from indicator development to indicator implementation at the MS level.

With the termination of the last ECHI project in 2012, however, there is no vested procedure in place to change, add or remove ECHI-indicators when health policy needs change, better data become available or data sources deteriorate. Changes in underlying data sources call for timely and thorough updates of the ECHI-shortlist and its metadata, following the manner of the structured documentation sheets that were developed in the JA-ECHIM. Accordingly, indicators which take data from the EHIS have to be examined against latest EHIS developments. Is the chosen operationalisation adequate or somewhat deviating from the original definitions? Also, there is currently no regular inventory of problems met by the MS in collecting the necessary data timely and with sufficient quality and comparability. The last JA-ECHIM recommended that an update be applied preferably every 3 years, but already anticipated that the future was uncertain, e.g. that the ECHIM Core Group might no longer exist. We now know this has become true, just as websites of relevant and related projects have been discontinued (e.g., echim.org; euphix.org). This lack of continuity is a real problem that cannot be countered by adding new projects to the history of ECHI. We need a sustainable new governance structure for the ECHI and for EU health information in general.

Our surveys, conducted in the context of the BRIDGE Health project, show a continued need to work on aspects of implementation, but also a need for renewed/increased attention for content aspects such as relevance for policy (priorities) and actionability. The experts agree that suggestions for new policy areas will need to go through a structured procedure, which needs to be developed.

### A possible future for ECHI

The audience and needs for ECHI are complex. The indicator list needs to be relatively short and actionable in the view of policy makers, but provide for more (detailed) information - not just for researchers, but also for policy makers - when a change in indicator outcome is signaled.

A well-organised ECHI-process may support priority setting in health policy and may also show where investment in data collection and indicator development is needed. At the same time, the fact that policy priorities often shift needs to be handled as well. A future implementation of the ECHI will provide continuous opportunities to discuss and evaluate current national health trends against developments in other European countries, thereby facilitating an exchange about measures taken in prevention and care. Challenges ahead for the ECHI are to increase data availability to reduce health information inequalities in Europe. A major issue to tackle is how to organise sustainable governance for the ECHI process. Addressing comparability and quality issues and having high quality meta-information remains highly important. An indicator repository aiming to make ECHI-related (meta-) information available for researchers, policymakers and the interested public in a more sustainable way is useful to increase ECHI visibility and use.

## Conclusion

In summary, our evaluations suggest that there is a need to invest in a continuous and collaborative effort from EU MS to:

- Strengthen the links between the ECHI -shortlist and policy makers and policy priorities.
- Further develop the ECHI format, i.e., to develop layering or sections to more adequately address the need for both stability and flexibility, also taking into account a suitable size and accommodate both the need for general monitoring and actionability by defining specific policy targets and commitments.
- Organise a structured procedure to identify new areas of health policy information for the EU and its MS.
- Evaluate how to improve the role of health systems performance in ECHI, e.g. by evaluating (when available) results from the European Health System Indicator survey that was performed by partners of the BRIDGE Health project, which is aimed at harmonising monitoring of health systems and health policy.
- Develop a structured procedure to maintain and update the ECHI process, including sustainable governance.
- Establish an ECHI indicator platform, i.e. a single point of access for
  - Easy and sustainable access to existing methodologies, expertise, historical and current knowledge; an important aspect here is that this platform may link through to other websites and indicators, i.e. fulfil a pointer function, where possible, in order to be more efficient. This will also contribute to visualising the place the ECHI have in the overarching European health information landscape.
  - Exchange of expertise and capacity building on health indicators and their use in EU.
  - And possibly also facilitating a structural mechanism for updating the ECHI meta-data, both content-wise and technical.
- Actively promote and evaluate the use of ECHI, as using the data will teach us valuable lessons. We call out to the research and policy communities to report on the concrete use of ECHI and resulting policy lessons.
- Develop joint projects and data collections with the major international organisations active in the European region, to efficiently and sustainably embed ECHI in the international health information landscape.

Combined with recommendations and issues identified in earlier evaluations, we conclude that there is a good knowledge base that can be used to improve, expand, adapt, reduce and focus the ECHI shortlist in the future.

Furthermore, there is a general positive consensus among stakeholders for having a permanent health indicator system like the ECHI at the European level, particularly under a clearer institutional and legal framework, and also including other international organisations and institutions, such as WHO/Euro, WHO and Eurostat [16, 20]. WP4 of the BRIDGE Health project has made a new start for ECHI after the previous ECHI-project ended in 2012, by assessing data availability and content-related aspects, of which the first results are presented here. The work will continue under the Joint Action on Health Information (2018–2021), with special attention for policy prioritisation and sustainable update procedures. The methods and infrastructures developed within the larger context of the Joint Action will become part of a sustainable health information system at European level.

## Additional file

Additional file 1: **Table S1.** Proportion of countries with available / unavailable / missing data in the preferred international data source (implementation section/*n* = 67). **Table S2.** Proportion of countries with available / unavailable / missing data in the preferred international data source (work-in-progress section/*n* = 5). (DOCX 21 kb)

## Abbreviations

ACG: Advisory Core Group; expert group established under work package 4 of BRIDGE Health with representatives of Eurostat, OECD, WHO and/or academia in the field of public health
AMI: Acute Myocardial Infarction
BRIDGE Health: BRidging Information and Data Generation for Evidence-based Health policy and research; the BRIDGE Health project aimed to prepare the transition towards a sustainable and integrated EU health information system for both public health and research purposes. The project was launched in May 2015 and ran for 30 months until October 2017. It included 31 partners in 16 countries
DG EMPL: Directorate General for Employment, Social Affairs & Inclusion
DG RTD: Directorate General for Research and Innovation
DG SANTE: Directorate General for Health and Food Safety
EC: European commission
ECHI: European Core Health Indicators, the shortlist contains 88 indicators mostly derived from EUROSTAT, WHO and OECD data sources, available for most Member States of the European Union, developed between 1998 and 2013
ECHI-1: European Community Health Indicators, phase 1, EU-funded project between 1998 and 2001, developed a 1st version of the ECHI longlist
ECHI-2: European Community Health Indicators, phase 2, EU-funded project between 2003 and 2005, developed an ECHI extended longlist and extracted 82 indicators for the ECHI shortlist
ECHIM: European Community Health Indicators Monitoring, EU-funded project between 2005 and 2008, update of ECHI shortlist (88 indicators), aimed at developing and implementing European Community Health Indicators (ECHI) and a population-based health monitoring system in the European Union
EFTA: European Free Trade Association
EGHI: EU Expert Group on Health Information; advisory group of representatives from EU MS, European Economic Area countries, possible future MS and international organisations, helping EU MS to make evidence-based health policy
EG-NHII: Expert Group on National Health Indicator Implementation; established under work package 4 of BRIDGE Health with over 20 members of the EU Expert Group on Health Information (EGHI)
EHIS: European Health Interview Survey, first wave was conducted between 2006 and 2009 (17 EU MS), a second – from then on mandatory – wave was conducted between 2013 and 2015 in all EU MS, a third wave is planned for 2019. EHIS is an important data source for the ECHI
EMCDDA: European Monitoring Centre for Drugs and Drug Addiction
EU: European Union
euHS_I survey: european Health System Indicator survey, conducted by WP12 (Evaluation of health care systems) and the Health System Indicator Task Force of the BRIDGE Health project with the objective to establish a consensus on a set of indicators which are of greatest value for Health System Performance Assessment (HSPA)
EURO-PERISTAT: EURO-PERISTAT began in 1999 with the goal to develop indicators for monitoring and evaluating perinatal health in the EU. EURO-PERISTAT releases the European Perinatal Health Report
EUROSTAT: The European Union’s statistical office, DG ESTAT
EU-SILC: EU-Statistics on Income and Living Conditions, providing annual data since 2004 for 15 EU countries, and since 2005 for all EU MS, Norway and Iceland, now also including Switzerland and Turkey based on a common framework
HBSC: Study on Health Behaviour in School Aged Children; research collaboration with the WHO Regional Office for Europe, the study is conducted every 4 years
HFA-DB: European Health for All Database with indicators relevant to the WHO EU-Region
JA-ECHIM: Joint Action for European Community Health Indicators Monitoring, EU-funded project between 2008 and 2012, building on EHIM, aiming to develop more precise definitions of the ECHI indicators and continued the implementation in the MS
JAF Health: Joint Assessment Framework on Health, EU Social Protection Committee, Indicators’ Sub-group
MS: Member States
OECD: Organisation for Economic Co-operation and Development
PHEIAC: Public Health Evaluation and Impact Assessment Consortium, a research consortium under the lead of Economisti Associati, an Italy based independent consulting firm
RIVM: National Institute for Public Health and the Environment in the Netherlands
RKI: Robert Koch Institute; Public Health Institute of Germany
TAIEX: Technical Assistance and Information Exchange Instrument of the European Commission
WHO: World Health Organisation
WP: Work package
