# Supplementary material for: European Core Health Indicators - status and perspectives
Source: Arch Public Health. 2018 Aug 3;76:52. doi: 10.1186/s13690-018-0298-9 (PMC6080502; doi:10.1186/s13690-018-0298-9)
Supplement: Supplementary file 1 — Table S1. Proportion of countries with available / unavailable / missing data in the preferred international data source (implementation section/n = 67). Table S2. Proportion of countries with available / unavailable / missing data in the preferred international data source (work-in-progress section/n = 5). (DOCX 21 kb) [file 13690_2018_298_MOESM1_ESM.docx]

**Additional file 1**

Table S1: Proportion of countries with available / unavailable / missing data in the preferred international data source (implementation section/n=67)**

| **ECHI Shortlist – Implementation Section** | **Data available (%)** | **No data available (%)** | **Missing (%)** |
| --- | --- | --- | --- |
| **Demography and socioeconomic situation** |  |  |  |
| 1. Population by sex/age | 100 | 0 | 0 |
| 2. Birth rate, crude | 100 | 0 | 0 |
| 4. Total fertility rate | 100 | 0 | 0 |
| 5. Population projections | 100 | 0 | 0 |
| 7. Population by occupation | 100 | 0 | 0 |
| 8. Total unemployment | 100 | 0 | 0 |
| 6. Population by education | 96 | 0 | 4 |
| 9. Population below poverty line and income inequality | 96 | 0 | 4 |
| 3. Mother’s age distribution | 91 | 9 | 0 |
| **Health status** |  |  |  |
| 10. Life expectancy | 100 | 0 | 0 |
| 11. Infant mortality | 100 | 0 | 0 |
| 12. Perinatal mortality | 100 | 0 | 0 |
| 18. Selected communicable diseases | 100 | 0 | 0 |
| 19. HIV/AIDS | 100 | 0 | 0 |
| 20. Cancer incidence | 100 | 0 | 0 |
| 28. (Low) birth weight | 100 | 0 | 0 |
| 13. Disease-specific mortality | 96 | 0 | 4 |
| 14. Drug-related deaths | 96 | 0 | 4 |
| 30. (B) Injuries: road traffic, register-based incidence | 96 | 0 | 4 |
| 31. Injuries: workplace | 96 | 0 | 4 |
| 33. Self-perceived health | 96 | 4 | 0 |
| 34. Self-reported chronic morbidity | 96 | 4 | 0 |
| 35. Long-term activity limitations | 96 | 4 | 0 |
| 40. Health expectancy: HLY | 96 | 0 | 4 |
| 21. (A) Diabetes, self-reported prevalence | 87 | 13 | 0 |
| 23. (A) Depression, self-reported prevalence | 87 | 13 | 0 |
| 26. (A) Asthma, self-reported prevalence | 87 | 13 | 0 |
| 36. Physical and sensory functional limitations | 87 | 13 | 0 |
| 27. (A) COPD, self-reported prevalence | 83 | 17 | 0 |
| 29. (A) Injuries: home/leisure, violence, self-reported incidence | 78 | 17 | 4 |
| 30. (A) Injuries: road traffic, self-reported incidence | 78 | 17 | 4 |
| 29. (B) Injuries: home/leisure, violence, register-based incidence | 52 | 43 | 4 |
| **Determinants of health** |  |  |  |
| 46. Total alcohol consumption | 100 | 0 | 0 |
| 55. PM10 (particulate matter) exposure | 100 | 0 | 0 |
| 53. Work-related health risks | 96 | 4 | 0 |
| 44. Regular smokers | 91 | 9 | 0 |
| 48. Use of illicit drugs | 91 | 0 | 9 |
| 47. Hazardous alcohol consumption | 87 | 4 | 9 |
| 49. Consumption of fruit | 87 | 9 | 4 |
| 50. Consumption of vegetables | 87 | 9 | 4 |
| 52. Physical activity | 87 | 9 | 4 |
| 54. Social support | 87 | 9 | 4 |
| 42. Body mass index | 83 | 9 | 9 |
| 43. Blood pressure | 83 | 9 | 9 |
| **Health services** |  |  |  |
| 56. Vaccination coverage in children | 100 | 0 | 0 |
| 62. Hospital beds | 100 | 0 | 0 |
| 58. Breast cancer screening | 96 | 4 | 0 |
| 59. Cervical cancer screening | 96 | 4 | 0 |
| 63. Practising physicians | 96 | 4 | 0 |
| 70. Average length of stay (ALOS), limited diagnoses | 96 | 4 | 0 |
| 71. General practitioner (GP) utilisation | 96 | 4 | 0 |
| 72. Selected outpatient visits | 96 | 4 | 0 |
| 76. Insurance coverage | 96 | 4 | 0 |
| 80. Equity of access to health care services | 96 | 0 | 4 |
| 57. Influenza vaccination rate in elderly | 91 | 9 | 0 |
| 67. Hospital in-patient discharges, limited diagnoses | 91 | 9 | 0 |
| 73. Surgeries: PTCA, hip, cataract | 91 | 9 | 0 |
| 77. Expenditures on health | 91 | 9 | 0 |
| 60. Colon cancer screening | 87 | 13 | 0 |
| 68. Hospital daycases, limited diagnoses | 87 | 13 | 0 |
| 69. Hospital day-cases as percentage of total patient population | 87 | 13 | 0 |
| 66. Medical technologies: MRI units and CT scans | 83 | 17 | 0 |
| 74. Medicine use, selected groups | 83 | 13 | 4 |
| 64. Practising nurses | 78 | 22 | 0 |
| 78. Survival rates cancer | 70 | 30 | 0 |
| 79. 30-day in-hospital case-fatality AMI and stroke | 70 | 26 | 4 |
| **Health promotion** |  |  |  |
| 85. Policies on ETS exposure (Environmental Tobacco Smoke) | 100 | 0 | 0 |

Table S2: Proportion of countries with available / unavailable /missing data in the preferred international data source (work-in-progress section/n=5)**

| **ECHI shortlist indicators – Work in progress-section*** | **Data available (%)** | **No data available (%)** | **Missing (%)** |
| --- | --- | --- | --- |
| **Health status** |  |  |  |
| 15. Smoking-related deaths | 91 | 4 | 4 |
| 16. Alcohol-related deaths | 91 | 4 | 4 |
| 41. Health expectancy, others | 91 | 4 | 4 |
| **Determinants of health** |  |  |  |
| 51. Breastfeeding | 65 | 35 | 0 |
| **Health services** |  |  |  |
| 75. Patient mobility | 35 | 17 | 48 |

* Table 2 contains those 5 of the 14 indicators in the ECHI work-in-progress section which already have a defined preferred international data source; for the remaining 9 indicators, such data source has not been defined yet so data availability was not mapped for these indicators.

**Sum of percent distributions may not always equal to 100% because of rounding.
